# 3-Deoxy­aconitine from the root of *Aconitum Carmichaeli* Debx.

**DOI:** 10.1107/S1600536810016922

**Published:** 2010-05-15

**Authors:** Feng Gao, Shou-An Zhu, Shi-Jun Xiong

**Affiliations:** a Agromomy College, Sichuan Agriculture University, Yaan 625014, People’s Republic of China

## Abstract

The title compound (systematic name: 8β-acet­oxy-14α-benzo­yloxy-*N*-ethyl-13β,15α-dihydr­oxy-1α,6α,16β-trimeth­oxy-4β-methoxy­methyl­eneaconitane), C_34_H_47_NO_10_, is a typical aconitine-type C_19_-diterpenoid alkaloid, and was isolated from the roots of the *Aconitum carmichaeli* Debx. The mol­ecule has an aconitine carbon skeleton with four six-membered rings and two five-membered rings, whose geometry is similar to these observed in other C_19_-diterpenoid alkaloids; both of five-membered rings have the envelope configurations and the six-membered N-containing heterocyclic ring displays a chair conformation. Intra­molecular O—H⋯O hydrogen bonding occurs. Weak inter­molecular C—H⋯O hydrogen bonding is observed in the crystal structure.

## Related literature

The title compound is a C_19_-diterpenoid alkaloid: for a review of diterpenoid alkaloids, see Wang *et al.* (2009 ▶, 2010 ▶). For the chemical structure of the title compound established from NMR and MS data, see: Pelletier *et al.* (1984 ▶). For the structures of related C_19_-diterpenoid alkaloids, see: Tashkhodjaev & Sultankhodjaev (2009 ▶); He *et al.* (2008 ▶).
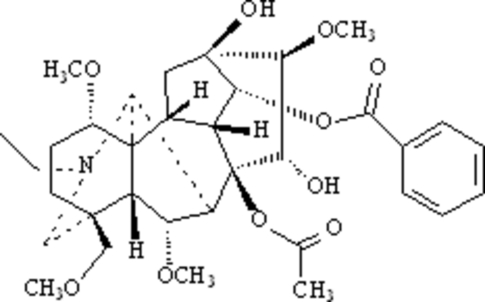


## Experimental

### 

#### Crystal data

C_34_H_47_NO_10_
*M* *_r_* = 629.73
Orthorhombic, 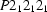

*a* = 12.039 (3) Å
*b* = 15.805 (4) Å
*c* = 17.320 (3) Å
*V* = 3295.5 (13) Å^3^
*Z* = 4
Mo *K*α radiation
μ = 0.09 mm^−1^
*T* = 292 K
0.58 × 0.52 × 0.42 mm

#### Data collection

Enraf–Nonius CAD-4 diffractometer
3323 measured reflections
3317 independent reflections
1669 reflections with *I* > 2σ(*I*)
*R*_int_ = 0.006
3 standard reflections every 300 reflections  intensity decay: 3.3%

#### Refinement

*R*[*F* ^2^ > 2σ(*F* ^2^)] = 0.076
*wR*(*F* ^2^) = 0.245
*S* = 0.97
3317 reflections
395 parameters
3 restraints
H-atom parameters constrained
Δρ_max_ = 0.64 e Å^−3^
Δρ_min_ = −0.42 e Å^−3^

### 

Data collection: *DIFRAC* (Flack *et al.*, 1992 ▶); cell refinement: *DIFRAC*; data reduction: *NRCVAX* (Gabe *et al.*, 1989 ▶); program(s) used to solve structure: *SHELXS97* (Sheldrick, 2008 ▶); program(s) used to refine structure: *SHELXL97* (Sheldrick, 2008 ▶); molecular graphics: *ORTEP-3 for Windows* (Farrugia, 1997 ▶); software used to prepare material for publication: *WinGX* (Farrugia, 1999 ▶).

## Supplementary Material

Crystal structure: contains datablocks I, global. DOI: 10.1107/S1600536810016922/xu2759sup1.cif

Structure factors: contains datablocks I. DOI: 10.1107/S1600536810016922/xu2759Isup2.hkl

Additional supplementary materials:  crystallographic information; 3D view; checkCIF report

## Figures and Tables

**Table 1:** Hydrogen-bond geometry (Å, °)

*D*—H⋯*A*	*D*—H	H⋯*A*	*D*⋯*A*	*D*—H⋯*A*
O4—H4*O*⋯O6	0.86	2.03	2.563 (9)	120
O5—H5*O*⋯O10	0.88	2.12	2.792 (8)	133
C31—H31⋯O1^i^	0.93	2.59	3.508 (12)	171

Symmetry code: (i) 
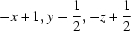
.

**Fig. 1.**
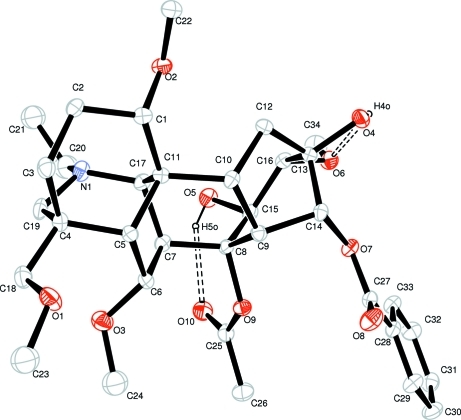
The molecular structure of the title compound with 30% probability displacement ellipsoids for non-H atoms. H atoms not including in hydrogen bonding have been omitted for clarity.

## supplementary crystallographic information

### Comment

The plant belonging to the genus *A. carmichaeli *Debx., which has been therapeutically used to treat rheumatic pain, paralysis due tostroke, rheumatoid arthritis and some other inflammations. The diterpenoid alkaloid, 3-deoxyaconitine, was previously isolated from *A. carmichaeli* Debx. (Pelletier *et al.* 1984), and its structure was established from the NMR and MS data. In our recent investigation, it was isolated from *A. carmichaeli* Debx., and its crystal structure was determined. The naming and the rings conforming referred to the literature (He *et al.*, 2008). The molecular structure of the title compound is shown in Fig. 1. Six-membered rings A (C1/C2/C3/C4/C5/C11) and B (C7/C8/C9/C10/C11/C17) adopt chair conformations; six-membered heterocyclic ring E (C4/C5/C11/C17/N1/C19) adopt the same chair conformation; the five-membered rings C (C9/C10/C12/C13/C14) and F (C5/C6/C7/C17/C11) display an envelope conformation. while the six-membered ring D (C8/C9/C14/C13/C16/C15) adopt boat conformations. The crystal structure contains intermolecular O—H···O hydrogen bond between the hydroxy group and carbonyl O atom. The absolute configuration of the title compound can not confirmed by the MoKa diffraction data. But it could be determined throngh the comparion of the similar natural products for their unique and same configuration (Tashkhodjaev *et al.*, 2009).

### Experimental

Air-dried and powdered roots (400 g) were percolated with 0.1 M HCl solution (5 L). The obtained acid aqueous solution was basified with 10% aqueous NH_4_OH to pH 10 and then extracted with ethyl acetate (6 L×3). Removal of the solvent under reduced pressure afforded the total crude alkaloids (2.0 g) as a yellowish amorphous powder, which was chromatographed over a silica gel column, eluting with cyclohexane-acetone (9:1→1:2) gradient system, to afford deoxyaconitine (180 mg) in cyclohexane-acetone (7:1) gradient system. The crystals suitable for X-ray structure analysis were obtained by slow evaporation from an acetone solution at room temperature.

### Refinement

Hydroxy H atoms were located in a difference Fourier map and refined as riding in their as-found relative positions with U_iso_(H) = 1.5U_eq_(O). Other H atoms were located geometrically with C—H = 0.93–0.98 Å, and refined using a riding model with U_iso_(H) = 1.5U_eq_(C) for methyl and 1.2U_eq_(C) for the others. The absolute configuration has not been determined from the X-ray analysis, owing to the absence of strong anomalous scattering, and Friedel's pairs were merged. Bond distance restraints for three bonds were applied.

### Crystal data

**Table:**

C_34_H_47_NO_10_	*F*(000) = 1352
*M**_r_* = 629.73	*D*_x_ = 1.269 Mg m^−^^3^
Orthorhombic, *P*2_1_2_1_2_1_	Mo *K*α radiation, λ = 0.71073 Å
Hall symbol: P 2ac 2ab	Cell parameters from 25 reflections
*a* = 12.039 (3) Å	θ = 4.3–7.6°
*b* = 15.805 (4) Å	µ = 0.09 mm^−^^1^
*c* = 17.320 (3) Å	*T* = 292 K
*V* = 3295.5 (13) Å^3^	Block, colourless
*Z* = 4	0.58 × 0.52 × 0.42 mm

### Data collection

**Table:**

Enraf–Nonius CAD-4 diffractometer	*R*_int_ = 0.006
Radiation source: fine-focus sealed tube	θ_max_ = 25.2°, θ_min_ = 1.7°
graphite	*h* = −1→14
ω/2θ scans	*k* = −5→18
3323 measured reflections	*l* = −2→20
3317 independent reflections	3 standard reflections every 300 reflections
1669 reflections with *I* > 2σ(*I*)	intensity decay: 3.3%

### Refinement

**Table:**

Refinement on *F*^2^	Primary atom site location: structure-invariant direct methods
Least-squares matrix: full	Secondary atom site location: difference Fourier map
*R*[*F*^2^ > 2σ(*F*^2^)] = 0.076	Hydrogen site location: inferred from neighbouring sites
*wR*(*F*^2^) = 0.245	H-atom parameters constrained
*S* = 0.97	*w* = 1/[σ^2^(*F*_o_^2^) + (0.1607*P*)^2^] where *P* = (*F*_o_^2^ + 2*F*_c_^2^)/3
3317 reflections	(Δ/σ)_max_ = 0.003
395 parameters	Δρ_max_ = 0.64 e Å^−^^3^
3 restraints	Δρ_min_ = −0.42 e Å^−^^3^

### Special details

**Table:**

Experimental. The absolute structure of the title compound can not be determined by the X-ray analysis, owing to the absence of strong anomalous scatterers. But it can be deduced by the comparision to the known diterpenoid alkaloids, for the unique absolute configuration of the similar natural products.
Geometry. All esds (except the esd in the dihedral angle between two l.s. planes) are estimated using the full covariance matrix. The cell esds are taken into account individually in the estimation of esds in distances, angles and torsion angles; correlations between esds in cell parameters are only used when they are defined by crystal symmetry. An approximate (isotropic) treatment of cell esds is used for estimating esds involving l.s. planes.
Refinement. Refinement of *F*^2^ against ALL reflections. The weighted *R*-factor wR and goodness of fit *S* are based on *F*^2^, conventional *R*-factors *R* are based on *F*, with *F* set to zero for negative *F*^2^. The threshold expression of *F*^2^ > σ(*F*^2^) is used only for calculating *R*-factors(gt) etc. and is not relevant to the choice of reflections for refinement. *R*-factors based on *F*^2^ are statistically about twice as large as those based on *F*, and *R*- factors based on ALL data will be even larger.

### Fractional atomic coordinates and isotropic or equivalent isotropic displacement parameters (Å^2^)

**Table:**

	*x*	*y*	*z*	*U*_iso_*/*U*_eq_	
O1	0.0042 (5)	0.5837 (4)	0.1632 (4)	0.098 (2)	
O2	0.3230 (5)	0.6590 (3)	−0.0985 (3)	0.0716 (15)	
O3	0.1594 (6)	0.4053 (4)	0.1289 (4)	0.0941 (19)	
O4	0.7008 (5)	0.6091 (4)	0.0024 (3)	0.0801 (16)	
H4O	0.7475	0.5761	−0.0195	0.120*	
O5	0.4878 (5)	0.3582 (3)	−0.0478 (3)	0.0765 (16)	
H5O	0.4653	0.3089	−0.0309	0.115*	
O6	0.7160 (5)	0.4502 (4)	−0.0251 (3)	0.0810 (16)	
O7	0.6524 (4)	0.4866 (3)	0.1332 (3)	0.0657 (13)	
O8	0.6094 (6)	0.5188 (4)	0.2555 (4)	0.097 (2)	
O9	0.4517 (4)	0.3843 (3)	0.1433 (3)	0.0662 (14)	
O10	0.4394 (6)	0.2592 (3)	0.0814 (4)	0.0903 (18)	
N1	0.1910 (6)	0.4752 (4)	−0.0645 (4)	0.0707 (18)	
C1	0.2542 (6)	0.6538 (4)	−0.0315 (4)	0.0600 (18)	
H1	0.2678	0.7044	−0.0002	0.072*	
C2	0.1342 (6)	0.6562 (5)	−0.0579 (5)	0.072 (2)	
H2A	0.1243	0.6177	−0.1010	0.086*	
H2B	0.1159	0.7128	−0.0754	0.086*	
C3	0.0569 (7)	0.6311 (5)	0.0073 (6)	0.079 (2)	
H3A	0.0601	0.6742	0.0472	0.095*	
H3B	−0.0186	0.6295	−0.0121	0.095*	
C4	0.0844 (6)	0.5465 (5)	0.0428 (4)	0.067 (2)	
C5	0.2014 (6)	0.5512 (5)	0.0806 (4)	0.0605 (18)	
H5	0.2018	0.5931	0.1223	0.073*	
C6	0.2419 (6)	0.4646 (5)	0.1107 (5)	0.065 (2)	
H6	0.2873	0.4742	0.1569	0.078*	
C7	0.3190 (6)	0.4311 (5)	0.0448 (4)	0.0615 (19)	
H7	0.3003	0.3724	0.0319	0.074*	
C8	0.4374 (6)	0.4373 (4)	0.0720 (4)	0.0560 (17)	
C9	0.4578 (6)	0.5269 (4)	0.1040 (4)	0.0593 (18)	
H9	0.4291	0.5311	0.1568	0.071*	
C10	0.4051 (6)	0.5967 (4)	0.0526 (4)	0.0573 (18)	
H10	0.3966	0.6474	0.0846	0.069*	
C11	0.2878 (6)	0.5758 (4)	0.0169 (4)	0.0528 (17)	
C12	0.4988 (6)	0.6149 (4)	−0.0071 (4)	0.0627 (19)	
H12A	0.4753	0.5985	−0.0586	0.075*	
H12B	0.5171	0.6747	−0.0075	0.075*	
C13	0.5991 (6)	0.5623 (5)	0.0184 (4)	0.0603 (18)	
C14	0.5799 (6)	0.5508 (5)	0.1023 (4)	0.0607 (19)	
H14	0.5914	0.6044	0.1295	0.073*	
C15	0.5311 (6)	0.4083 (5)	0.0126 (4)	0.0616 (19)	
H15	0.5822	0.3719	0.0416	0.074*	
C16	0.6017 (7)	0.4775 (5)	−0.0235 (4)	0.066 (2)	
H16	0.5767	0.4863	−0.0767	0.079*	
C17	0.2933 (7)	0.4900 (4)	−0.0239 (4)	0.0599 (19)	
H17	0.3556	0.4894	−0.0604	0.072*	
C18	−0.0072 (7)	0.5272 (5)	0.1004 (5)	0.081 (2)	
H18A	−0.0794	0.5344	0.0763	0.097*	
H18B	−0.0011	0.4693	0.1183	0.097*	
C19	0.0878 (7)	0.4761 (5)	−0.0181 (5)	0.080 (2)	
H19A	0.0801	0.4219	0.0077	0.096*	
H19B	0.0249	0.4827	−0.0525	0.096*	
C20	0.2009 (12)	0.4007 (9)	−0.1144 (6)	0.143 (2)	
H20A	0.1567	0.3551	−0.0929	0.172*	
H20B	0.2778	0.3823	−0.1151	0.172*	
C21	0.1628 (12)	0.4177 (9)	−0.1978 (6)	0.143 (2)	
H21A	0.0932	0.4473	−0.1971	0.215*	
H21B	0.1539	0.3648	−0.2245	0.215*	
H21C	0.2175	0.4514	−0.2239	0.215*	
C22	0.3336 (8)	0.7427 (5)	−0.1300 (5)	0.084 (2)	
H22A	0.2661	0.7578	−0.1561	0.126*	
H22B	0.3943	0.7439	−0.1659	0.126*	
H22C	0.3474	0.7822	−0.0890	0.126*	
C23	−0.0678 (9)	0.5696 (9)	0.2290 (5)	0.143 (2)	
H23A	−0.1428	0.5838	0.2154	0.215*	
H23B	−0.0440	0.6043	0.2713	0.215*	
H23C	−0.0643	0.5111	0.2438	0.215*	
C24	0.1575 (13)	0.3699 (8)	0.2060 (5)	0.143 (2)	
H24A	0.2245	0.3851	0.2327	0.215*	
H24B	0.1523	0.3093	0.2028	0.215*	
H24C	0.0945	0.3916	0.2336	0.215*	
C25	0.4466 (7)	0.2997 (5)	0.1407 (6)	0.076 (2)	
C26	0.4554 (10)	0.2620 (6)	0.2188 (5)	0.106 (3)	
H26A	0.4266	0.2054	0.2178	0.159*	
H26B	0.4133	0.2954	0.2547	0.159*	
H26C	0.5319	0.2609	0.2344	0.159*	
C27	0.6552 (7)	0.4755 (5)	0.2098 (5)	0.071 (2)	
C28	0.7279 (7)	0.4016 (5)	0.2299 (4)	0.066 (2)	
C29	0.7361 (9)	0.3785 (6)	0.3065 (5)	0.086 (3)	
H29	0.6988	0.4096	0.3440	0.104*	
C30	0.8009 (9)	0.3079 (6)	0.3281 (6)	0.094 (3)	
H30	0.8066	0.2916	0.3796	0.113*	
C31	0.8551 (8)	0.2644 (6)	0.2714 (6)	0.087 (3)	
H31	0.8986	0.2180	0.2845	0.104*	
C32	0.8465 (9)	0.2878 (6)	0.1959 (5)	0.087 (3)	
H32	0.8846	0.2571	0.1585	0.104*	
C33	0.7841 (7)	0.3543 (5)	0.1741 (5)	0.073 (2)	
H33	0.7783	0.3686	0.1221	0.088*	
C34	0.7429 (9)	0.3915 (7)	−0.0847 (6)	0.107 (3)	
H34A	0.7078	0.3382	−0.0742	0.160*	
H34B	0.8220	0.3839	−0.0867	0.160*	
H34C	0.7171	0.4129	−0.1334	0.160*	

### Atomic displacement parameters (Å^2^)

**Table:**

	*U*^11^	*U*^22^	*U*^33^	*U*^12^	*U*^13^	*U*^23^
O1	0.082 (4)	0.111 (5)	0.101 (4)	−0.021 (4)	0.031 (4)	−0.007 (4)
O2	0.082 (4)	0.070 (3)	0.062 (3)	0.010 (3)	0.005 (3)	0.012 (3)
O3	0.094 (5)	0.080 (4)	0.107 (4)	−0.008 (4)	0.011 (4)	0.013 (4)
O4	0.060 (3)	0.085 (4)	0.095 (4)	−0.007 (3)	0.002 (3)	0.013 (3)
O5	0.088 (4)	0.066 (3)	0.076 (3)	0.003 (3)	−0.003 (3)	−0.022 (3)
O6	0.064 (4)	0.095 (4)	0.084 (4)	0.016 (3)	0.008 (3)	0.006 (3)
O7	0.067 (3)	0.075 (3)	0.055 (3)	0.010 (3)	−0.011 (2)	0.004 (3)
O8	0.113 (5)	0.107 (5)	0.071 (4)	0.035 (4)	−0.006 (4)	−0.024 (3)
O9	0.075 (4)	0.061 (3)	0.063 (3)	0.008 (3)	−0.001 (3)	0.005 (2)
O10	0.103 (5)	0.060 (3)	0.107 (4)	−0.002 (3)	−0.009 (4)	0.010 (3)
N1	0.084 (5)	0.056 (4)	0.072 (4)	−0.002 (4)	−0.013 (4)	−0.024 (3)
C1	0.070 (5)	0.054 (4)	0.056 (4)	0.009 (4)	0.005 (4)	−0.001 (3)
C2	0.062 (5)	0.077 (5)	0.076 (5)	0.011 (4)	−0.011 (4)	0.015 (4)
C3	0.051 (4)	0.073 (5)	0.112 (7)	0.008 (4)	−0.003 (5)	0.002 (5)
C4	0.053 (5)	0.076 (5)	0.074 (5)	−0.002 (4)	−0.004 (4)	0.000 (4)
C5	0.059 (4)	0.060 (4)	0.063 (4)	−0.004 (4)	−0.001 (4)	0.002 (4)
C6	0.061 (5)	0.063 (4)	0.070 (4)	−0.008 (4)	−0.003 (4)	0.007 (4)
C7	0.074 (5)	0.052 (4)	0.059 (4)	0.001 (4)	−0.007 (4)	−0.007 (3)
C8	0.059 (4)	0.053 (4)	0.057 (4)	0.000 (4)	−0.009 (4)	0.003 (3)
C9	0.062 (5)	0.066 (4)	0.050 (4)	0.002 (4)	0.000 (3)	0.003 (3)
C10	0.065 (5)	0.046 (4)	0.061 (4)	0.004 (4)	−0.011 (4)	−0.006 (3)
C11	0.061 (4)	0.048 (4)	0.049 (4)	0.001 (3)	0.004 (3)	−0.004 (3)
C12	0.067 (5)	0.052 (4)	0.069 (5)	−0.006 (4)	0.003 (4)	0.005 (4)
C13	0.059 (5)	0.063 (4)	0.060 (4)	−0.009 (4)	−0.001 (4)	0.012 (4)
C14	0.057 (5)	0.058 (4)	0.068 (4)	−0.003 (4)	−0.011 (4)	−0.009 (4)
C15	0.066 (5)	0.062 (4)	0.056 (4)	0.009 (4)	−0.008 (4)	−0.014 (4)
C16	0.072 (5)	0.073 (5)	0.052 (4)	0.011 (4)	−0.003 (4)	0.003 (4)
C17	0.071 (5)	0.054 (4)	0.055 (4)	0.005 (4)	−0.008 (4)	−0.006 (3)
C18	0.063 (5)	0.073 (5)	0.107 (7)	−0.009 (4)	−0.017 (5)	0.003 (5)
C19	0.063 (6)	0.077 (5)	0.100 (6)	−0.005 (4)	−0.032 (5)	−0.003 (5)
C20	0.156 (6)	0.139 (6)	0.135 (5)	−0.016 (5)	−0.006 (5)	0.005 (4)
C21	0.156 (6)	0.139 (6)	0.135 (5)	−0.016 (5)	−0.006 (5)	0.005 (4)
C22	0.084 (6)	0.089 (6)	0.078 (5)	−0.003 (5)	0.007 (5)	0.014 (5)
C23	0.156 (6)	0.139 (6)	0.135 (5)	−0.016 (5)	−0.006 (5)	0.005 (4)
C24	0.156 (6)	0.139 (6)	0.135 (5)	−0.016 (5)	−0.006 (5)	0.005 (4)
C25	0.068 (6)	0.060 (5)	0.099 (7)	0.001 (4)	−0.010 (5)	0.006 (5)
C26	0.131 (9)	0.080 (6)	0.107 (7)	0.007 (6)	−0.012 (7)	0.036 (6)
C27	0.072 (5)	0.070 (5)	0.070 (5)	−0.013 (5)	−0.017 (5)	0.001 (4)
C28	0.064 (5)	0.065 (5)	0.069 (5)	−0.001 (4)	−0.014 (4)	−0.003 (4)
C29	0.109 (7)	0.087 (6)	0.063 (5)	0.016 (6)	−0.014 (5)	−0.001 (4)
C30	0.122 (8)	0.080 (6)	0.079 (6)	0.008 (6)	−0.025 (6)	0.017 (5)
C31	0.082 (6)	0.073 (5)	0.105 (7)	0.007 (5)	−0.018 (6)	0.000 (6)
C32	0.101 (7)	0.077 (6)	0.083 (6)	0.013 (6)	−0.002 (5)	−0.004 (5)
C33	0.086 (6)	0.074 (5)	0.059 (4)	0.004 (5)	−0.012 (4)	−0.001 (4)
C34	0.098 (8)	0.099 (7)	0.124 (8)	0.025 (6)	0.026 (7)	−0.007 (7)

### Geometric parameters (Å, °)

**Table:**

O1—C18	1.413 (10)	C11—C17	1.531 (9)
O1—C23	1.449 (12)	C12—C13	1.531 (10)
O2—C1	1.427 (9)	C12—H12A	0.9700
O2—C22	1.437 (9)	C12—H12B	0.9700
O3—C6	1.401 (9)	C13—C14	1.482 (10)
O3—C24	1.448 (12)	C13—C16	1.525 (11)
O4—C13	1.457 (9)	C14—H14	0.9800
O4—H4O	0.8549	C15—C16	1.520 (11)
O5—C15	1.411 (8)	C15—H15	0.9800
O5—H5O	0.8753	C16—H16	0.9800
O6—C34	1.425 (11)	C17—H17	0.9800
O6—C16	1.442 (9)	C18—H18A	0.9700
O7—C27	1.338 (9)	C18—H18B	0.9700
O7—C14	1.442 (9)	C19—H19A	0.9700
O8—C27	1.183 (10)	C19—H19B	0.9700
O9—C25	1.340 (9)	C20—C21	1.539 (15)
O9—C8	1.501 (8)	C20—H20A	0.9700
O10—C25	1.213 (10)	C20—H20B	0.9700
N1—C17	1.437 (9)	C21—H21A	0.9600
N1—C20	1.466 (15)	C21—H21B	0.9600
N1—C19	1.480 (11)	C21—H21C	0.9600
C1—C2	1.516 (11)	C22—H22A	0.9600
C1—C11	1.544 (10)	C22—H22B	0.9600
C1—H1	0.9800	C22—H22C	0.9600
C2—C3	1.517 (11)	C23—H23A	0.9600
C2—H2A	0.9700	C23—H23B	0.9600
C2—H2B	0.9700	C23—H23C	0.9600
C3—C4	1.508 (11)	C24—H24A	0.9600
C3—H3A	0.9700	C24—H24B	0.9600
C3—H3B	0.9700	C24—H24C	0.9600
C4—C18	1.519 (11)	C25—C26	1.482 (12)
C4—C19	1.534 (12)	C26—H26A	0.9600
C4—C5	1.555 (11)	C26—H26B	0.9600
C5—C6	1.543 (10)	C26—H26C	0.9600
C5—C11	1.566 (10)	C27—C28	1.501 (12)
C5—H5	0.9800	C28—C29	1.379 (10)
C6—C7	1.565 (11)	C28—C33	1.397 (11)
C6—H6	0.9800	C29—C30	1.412 (12)
C7—C8	1.504 (10)	C29—H29	0.9300
C7—C17	1.542 (10)	C30—C31	1.365 (13)
C7—H7	0.9800	C30—H30	0.9300
C8—C9	1.541 (10)	C31—C32	1.363 (12)
C8—C15	1.594 (11)	C31—H31	0.9300
C9—C14	1.518 (10)	C32—C33	1.345 (12)
C9—C10	1.553 (9)	C32—H32	0.9300
C9—H9	0.9800	C33—H33	0.9300
C10—C12	1.557 (10)	C34—H34A	0.9600
C10—C11	1.577 (10)	C34—H34B	0.9600
C10—H10	0.9800	C34—H34C	0.9600
			
C18—O1—C23	116.7 (7)	O5—C15—C8	112.2 (6)
C1—O2—C22	114.4 (6)	C16—C15—C8	117.0 (6)
C6—O3—C24	118.5 (8)	O5—C15—H15	106.4
C13—O4—H4O	109.1	C16—C15—H15	106.4
C15—O5—H5O	111.5	C8—C15—H15	106.4
C34—O6—C16	115.2 (7)	O6—C16—C15	109.0 (6)
C27—O7—C14	118.3 (6)	O6—C16—C13	107.0 (6)
C25—O9—C8	121.5 (6)	C15—C16—C13	115.2 (6)
C17—N1—C20	110.4 (7)	O6—C16—H16	108.5
C17—N1—C19	116.9 (6)	C15—C16—H16	108.5
C20—N1—C19	113.3 (8)	C13—C16—H16	108.5
O2—C1—C2	107.8 (6)	N1—C17—C11	109.4 (6)
O2—C1—C11	109.5 (5)	N1—C17—C7	116.9 (6)
C2—C1—C11	115.7 (6)	C11—C17—C7	100.8 (5)
O2—C1—H1	107.9	N1—C17—H17	109.8
C2—C1—H1	107.9	C11—C17—H17	109.8
C11—C1—H1	107.9	C7—C17—H17	109.8
C1—C2—C3	110.7 (6)	O1—C18—C4	107.9 (6)
C1—C2—H2A	109.5	O1—C18—H18A	110.1
C3—C2—H2A	109.5	C4—C18—H18A	110.1
C1—C2—H2B	109.5	O1—C18—H18B	110.1
C3—C2—H2B	109.5	C4—C18—H18B	110.1
H2A—C2—H2B	108.1	H18A—C18—H18B	108.4
C4—C3—C2	113.6 (7)	N1—C19—C4	113.7 (6)
C4—C3—H3A	108.8	N1—C19—H19A	108.8
C2—C3—H3A	108.8	C4—C19—H19A	108.8
C4—C3—H3B	108.8	N1—C19—H19B	108.8
C2—C3—H3B	108.8	C4—C19—H19B	108.8
H3A—C3—H3B	107.7	H19A—C19—H19B	107.7
C3—C4—C18	106.6 (7)	N1—C20—C21	112.9 (11)
C3—C4—C19	111.7 (7)	N1—C20—H20A	109.0
C18—C4—C19	109.0 (7)	C21—C20—H20A	109.0
C3—C4—C5	109.2 (6)	N1—C20—H20B	109.0
C18—C4—C5	113.0 (6)	C21—C20—H20B	109.0
C19—C4—C5	107.4 (6)	H20A—C20—H20B	107.8
C6—C5—C4	112.8 (6)	C20—C21—H21A	109.5
C6—C5—C11	104.4 (6)	C20—C21—H21B	109.5
C4—C5—C11	108.5 (6)	H21A—C21—H21B	109.5
C6—C5—H5	110.3	C20—C21—H21C	109.5
C4—C5—H5	110.3	H21A—C21—H21C	109.5
C11—C5—H5	110.3	H21B—C21—H21C	109.5
O3—C6—C5	116.4 (6)	O2—C22—H22A	109.5
O3—C6—C7	110.9 (6)	O2—C22—H22B	109.5
C5—C6—C7	103.9 (6)	H22A—C22—H22B	109.5
O3—C6—H6	108.4	O2—C22—H22C	109.5
C5—C6—H6	108.4	H22A—C22—H22C	109.5
C7—C6—H6	108.4	H22B—C22—H22C	109.5
C8—C7—C17	113.1 (6)	O1—C23—H23A	109.5
C8—C7—C6	108.1 (6)	O1—C23—H23B	109.5
C17—C7—C6	103.9 (6)	H23A—C23—H23B	109.5
C8—C7—H7	110.5	O1—C23—H23C	109.5
C17—C7—H7	110.5	H23A—C23—H23C	109.5
C6—C7—H7	110.5	H23B—C23—H23C	109.5
O9—C8—C7	109.3 (6)	O3—C24—H24A	109.5
O9—C8—C9	101.4 (5)	O3—C24—H24B	109.5
C7—C8—C9	108.8 (6)	H24A—C24—H24B	109.5
O9—C8—C15	106.9 (5)	O3—C24—H24C	109.5
C7—C8—C15	116.7 (6)	H24A—C24—H24C	109.5
C9—C8—C15	112.6 (6)	H24B—C24—H24C	109.5
C14—C9—C8	112.0 (6)	O10—C25—O9	124.0 (8)
C14—C9—C10	102.0 (6)	O10—C25—C26	124.4 (8)
C8—C9—C10	112.4 (6)	O9—C25—C26	111.5 (8)
C14—C9—H9	110.1	C25—C26—H26A	109.5
C8—C9—H9	110.1	C25—C26—H26B	109.5
C10—C9—H9	110.1	H26A—C26—H26B	109.5
C9—C10—C12	102.5 (6)	C25—C26—H26C	109.5
C9—C10—C11	116.3 (6)	H26A—C26—H26C	109.5
C12—C10—C11	115.2 (5)	H26B—C26—H26C	109.5
C9—C10—H10	107.4	O8—C27—O7	125.2 (8)
C12—C10—H10	107.4	O8—C27—C28	124.5 (8)
C11—C10—H10	107.4	O7—C27—C28	110.2 (8)
C17—C11—C1	117.9 (5)	C29—C28—C33	119.2 (8)
C17—C11—C5	97.7 (6)	C29—C28—C27	118.1 (8)
C1—C11—C5	114.0 (6)	C33—C28—C27	122.6 (7)
C17—C11—C10	109.1 (6)	C28—C29—C30	120.3 (9)
C1—C11—C10	106.3 (5)	C28—C29—H29	119.8
C5—C11—C10	111.7 (5)	C30—C29—H29	119.8
C13—C12—C10	106.2 (5)	C31—C30—C29	118.0 (9)
C13—C12—H12A	110.5	C31—C30—H30	121.0
C10—C12—H12A	110.5	C29—C30—H30	121.0
C13—C12—H12B	110.5	C30—C31—C32	121.2 (9)
C10—C12—H12B	110.5	C30—C31—H31	119.4
H12A—C12—H12B	108.7	C32—C31—H31	119.4
O4—C13—C14	112.4 (6)	C33—C32—C31	121.6 (9)
O4—C13—C16	109.7 (6)	C33—C32—H32	119.2
C14—C13—C16	111.2 (6)	C31—C32—H32	119.2
O4—C13—C12	109.4 (6)	C32—C33—C28	119.6 (8)
C14—C13—C12	103.1 (6)	C32—C33—H33	120.2
C16—C13—C12	110.9 (6)	C28—C33—H33	120.2
O7—C14—C13	110.9 (6)	O6—C34—H34A	109.5
O7—C14—C9	113.9 (6)	O6—C34—H34B	109.5
C13—C14—C9	101.6 (6)	H34A—C34—H34B	109.5
O7—C14—H14	110.1	O6—C34—H34C	109.5
C13—C14—H14	110.1	H34A—C34—H34C	109.5
C9—C14—H14	110.1	H34B—C34—H34C	109.5
O5—C15—C16	107.8 (6)		

### Hydrogen-bond geometry (Å, °)

**Table:**

*D*—H···*A*	*D*—H	H···*A*	*D*···*A*	*D*—H···*A*
O4—H4O···O6	0.86	2.03	2.563 (9)	120
O5—H5O···O10	0.88	2.12	2.792 (8)	133
C31—H31···O1^i^	0.93	2.59	3.508 (12)	171

Symmetry codes: (i) −*x*+1, *y*−1/2, −*z*+1/2.

## References

[bb1] Farrugia, L. J. (1997). *J. Appl. Cryst.* **30**, 565.

[bb2] Farrugia, L. J. (1999). *J. Appl. Cryst.* **32**, 837–838.

[bb4] Flack, H. D., Blanc, E. & Schwarzenbach, D. (1992). *J. Appl. Cryst.* **25**, 455–459.

[bb5] Gabe, E. J., Le Page, Y., Charland, J.-P., Lee, F. L. & White, P. S. (1989). *J. Appl. Cryst.* **22**, 384–387.

[bb6] He, D.-H., Zhu, Y.-C. & Hu, A.-X. (2008). *Acta Cryst.* E**64**, o1033–o1034. 10.1107/S1600536808013147 PMC2961389 21202557

[bb7] Pelletier, S. W., Mody, N. V., Joshi, B. S. & Schramm, L. C. (1984). *The Alkaloids: Chemistry and Perspectives*, Vol. 2, edited by S. W. Pelletier, pp. 206–264. New York: Wiley.

[bb8] Sheldrick, G. M. (2008). *Acta Cryst.* A**64**, 112–122. 10.1107/S0108767307043930 18156677

[bb9] Tashkhodjaev, B. & Sultankhodjaev, M. N. (2009). *Acta Cryst.* E**65**, o1543–o1544. 10.1107/S1600536809021436 PMC2969206 21582830

[bb10] Wang, F.-P., Chen, Q.-H. & Liang, X.-T. (2009). *The Alkaloids: Chemistry and Biology*, Vol. 67, edited by G. A. Cordell, pp. 1–78. New York: Elsevier.

[bb11] Wang, F.-P., Chen, Q.-H. & Liu, X.-Y. (2010). *Nat. Prod. Rep.* **27**, 529–570. 10.1039/b916679c 20336236

